# Analytical Techniques and Pharmacokinetics of *Gastrodia elata* Blume and Its Constituents

**DOI:** 10.3390/molecules22071137

**Published:** 2017-07-08

**Authors:** Jinyi Wu, Bingchu Wu, Chunlan Tang, Jinshun Zhao

**Affiliations:** Department of Preventative Medicine, Zhejiang Provincial Key Laboratory of Pathological and Physiological Technology, Medical School of Ningbo University, Ningbo 315211, Zhejiang, China; wujiny@foxmail.com (J.W.); bingchu97@163.com (B.W.)

**Keywords:** *Gastrodia elata* Blume, analytical technique, pharmacokinetics, sample pretreatment

## Abstract

*Gastrodia elata* Blume (*G. elata*), commonly called Tianma in Chinese, is an important and notable traditional Chinese medicine (TCM), which has been used in China as an anticonvulsant, analgesic, sedative, anti-asthma, anti-immune drug since ancient times. The aim of this review is to provide an overview of the abundant efforts of scientists in developing analytical techniques and performing pharmacokinetic studies of *G. elata* and its constituents, including sample pretreatment methods, analytical techniques, absorption, distribution, metabolism, excretion (ADME) and influence factors to its pharmacokinetics. Based on the reported pharmacokinetic property data of *G. elata* and its constituents, it is hoped that more studies will focus on the development of rapid and sensitive analytical techniques, discovering new therapeutic uses and understanding the specific in vivo mechanisms of action of *G. elata* and its constituents from the pharmacokinetic viewpoint in the near future. The present review discusses analytical techniques and pharmacokinetics of *G. elata* and its constituents reported from 1985 onwards.

## 1. Introduction

*Gastrodia elata* Blume (*G. elata*), the tuber of the orchid, is considered a top grade herbal medicine, with a long history of use in many Asian countries for the treatment of many diseases thanks to its multiple activities, for example, anticonvulsion [1], antioxidation [2,3,4], anti-depression [5,6], analgesia [7], sedation [8,9], anti-epilepsy [10], anti-obesity [11], anti-asthma [12], anti-inflammation [13,14,15], learning and memory improvement [16,17,18] and neuroprotection [19,20,21,22].

**Figure 1:**
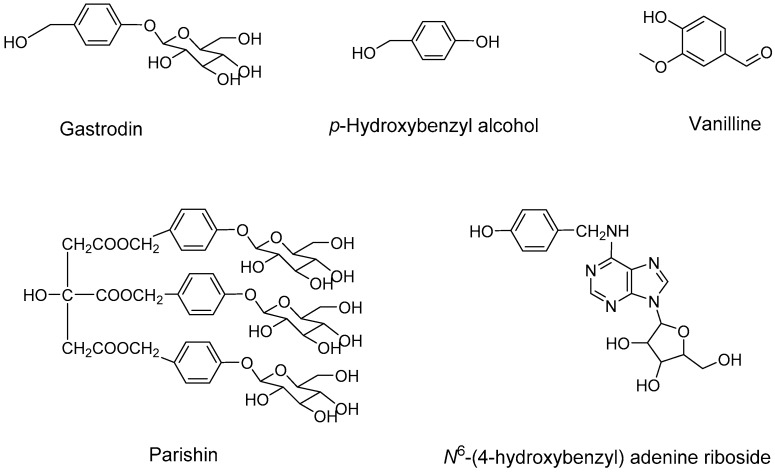
The structures of main bioactive constituents from *G. elata*.

The composition of *G. elata* is highly complex, mostly unknown and exhibits variation due to the planting, harvesting, storage and manufacturing processes used. Currently, over 81 compounds from *G. elata* have been isolated and identified, mainly phenolics, organic acids, sterols, and so forth. The structures of the main reported ingredients in *G. elata* with an influence on its pharmacokinetics (PK) are shown in Figure 1. Among them, GAS and its aglycone gastrodigenin (*p*-hydroxybenzyl alcohol, HBA) are considered as the characteristic and main active constituents of *G. elata* [20,23,24], and the recent Chinese Pharmacopeia (2015 version) added HBA as one of the phytochemical markers of *G. elata* [25], that is, the quality of *G. elata* is controlled by both GAS and HBA. It is well known that multiple components as well as some trace ingredients in herbal medicines are commonly administrated in clinical applications, and the constituents active in vivo are sometimes quite different from the pharmacologically active substances because of various biotransformations caused by phase I and phase II metabolic enzymes. Therefore, PK studies of TCMs have been a tremendous challenge and need sufficient technological support. An ideal PK assay for an herbal medicine should simultaneously qualitatively and quantitatively characterize “what are absorbed (the bioavailable phytochemical compounds)”, “what are produced (the new compounds produced through biotransformation)”, “how they are distributed (the compounds distributed by blood circulation)” and “how they are excreted (the excretion process of the compounds)”, or simply W_2_H_2_ for short.

In the past few decades, a large number of studies, including phytochemistry, pharmacokinetics, pharmacology, etc., on *G. elata* have been conducted, but only four reviews of *G. elata* are available. In the earliest review, Ojemann reviewed in 2006 the chemistry, pharmacology, animal data, and clinical use of *G. elata* and its constituents [10]. Then Chen and Sheen gave a short review about biological activities and antidepressant mechanisms of *G. elata* in 2011 [26], Jang et al. summarized the neuropharmacological potential of *G. elata* in 2015 [27], and Zhan et al. reviewed the botany and ethnopharmacology, phytochemistry, pharmacology, toxicology and quality control of *G. elata* in 2016 [28]. Little information about the biotransformation and pharmacokinetic properties of *G. elata* was available in the above reviews, so in this review, the abundant advances in sample pretreatment, separating and detecting techniques, absorption, distribution, metabolism and excretion (ADME) of *G. elata* and its constituents are summarized, and the possible trends and perspective for future investigation of *G. elata* are also put forward. This review covers the literature available from 1985 to 2016. The information was collected from scientific journals via a library and electronic search (using Baidu Scholar, PubMed, Web of Science, Science Direct and China National Knowledge Infrastructure).

## 2. Sample Pretreatment Method

It is well known that sample pretreatment is a significant step for the determination of compounds in biological samples. The biological sample pretreatment method for *G. elata* or its constituents included organic solvent precipitation, acid pretreatment and the solid phase extraction (SPE) method. Among them, the organic solvent precipitation method was the most commonly used method, attributing to its simple operation, high recovery and good repeatability.

### 2.1 Organic Solvent Precipitation

The solvents used to extract GAS, HBA, parishin, *N*^6^-(4-hydroxybenzyl) adenine riboside (NHBA) in biological samples have included ethanol [29,30], methanol [31,32,33,34], acetonitrile [35,36,37], *n*-butyl alcohol [38], methanol–acetonitrile (1:4) [39,40], aqueous ammonia–ether (1:4) [41], ethanol–dichloromethane [42], methanol-dichloromethane [43], diethyl ether [44] and ethyl acetate [45]. The volume of solvent used was about 3–5 fold greater than that of the samples. Among them, methanol and acetonitrile were the most common used for further LC analysis.

### 2.2. Acid Pretreatment

The acid pretreatment method is generally used to extract compounds in biological samples which are stable in an acidic system. Wang et al. investigated the pharmacokinetics and the kinetic inter-relationships of GAS. The plasma samples were handled using 6–8% perchloric acid for precipitation. The absolute recovery was at the range of 89.1–100.9%, which satisfied the requirements for determination of analytes in biological samples [46,47]. In addition, Lu et al. used perchlorate-30% hydrogen peroxide (1:2) and trichloroacetic acid for precipitation of blood, bile, urine, fecesand gastrointestinal tract samples [44]. These reports suggest that GAS is stable in an acidic system, but for extraction of other active constituents in *G. elata*, such as the analogues of parishin that are unstable in an acidic system, this method could not be employed.

### 2.3. Solid Phase Extraction

Lv et al. compared the methods of solvent extraction, protein precipitation and solid phase extraction to extract GAS, pointing out that the drying process after extraction caused a significant loss of the analytes after the solvent extraction method, and samples were not clean enough for MS/MS analysis using the protein precipitation method. Therefore, an Oasis HLB solid phase extraction column was used for the purification of plasma samples. This simple and rapid procedure produced a clean chromatogram for a blank plasma sample and yielded satisfactory recovery about 82% for analytes in plasma [48]. According to the previous method, our lab has compared the above method for simultaneously extracting the main phenolic constituents, GAS and parishin in plasma samples. Given the acid lability of parishin [49], only the organic solvent precipitation and solid phase extraction method were carried out. We added the GAS and parishin into the blank plasma, and used methanol, commercially available C18-SPE and home-made MCI-SPE to extract GAS and parishin. The results (supplementary material) showed that the MCI-SPE method was the best way for simultaneously extracting analytes with high recoveries and less interference. The methanol extraction gave a relative unclean analytical sample and the C18-SPE method retained less of GAS, so it might not be suitable for extraction of more polar metabolites of GAS or constituents in *G. elata*. The SPE method could provide samples clean enough for further LC or MS analysis, but for high-throughput experiments, this off-line SPE method might be quite time-consuming and lab intensive.

According the structures of compounds, acid stability and operability, we have determined that organic solvent precipitation was suited for high-throughput experimentation, otherwise the SPE method is an alternative.

## 3. Analytical Methods

The target of the pharmacokinetic study of herbal medicines is qualitative and quantitative characterizing “W_2_H_2_” in the global metabolite pool. A qualitatively and quantitatively analytical method is a prerequisite for biotransformation and pharmacokinetic analysis. Even though some good examples have been shown in the studies of herbal medicines [50,51], one can still imagine the difficulties and technical challenges to achieve this target because of the unknown, complex and variable composition of herbal medicines, as well as their various metabolic process in vivo. Currently, many separating and detecting techniques have been employed to evaluate the biotransformation and PK of *G. elata* and its constituents in biological samples, which are summarized as follows:

### 3.1. Liquid Chromatography (LC)

To date, the main reported method for separating *G. elata* or its constituents in biological systems, such as rat plasma [52], bile [53], intestine [54], liver [55], kidney [56], brain [45], cerebrospinal fluid [46], different brain regions of brain tissue homogenate [57], urine, feces [29], dog plasma [43], human plasma [42,58], rabbit plasma [59], is high performance liquid chromatography (HPLC), where reversed phase HPLC is the most widely employed separating mode compared to the normal phase mode. Various stationary phases are available and useful for the chromatographic separation of *G. elata* or its constituents. Gradient and isocratic elution were both carried out, and binary systems consisting of an aqueous system and a less polar organic solvent, such as acetonitrile or methanol, were the most common techniques. Moreover, a weak buffer, such as acetic acid or formic acid, has been used to maintain a suitable pH during gradient runs. Although HPLC is better than other techniques owing to its robustness and being easy to operate, the high resolution and efficiency of HPLC in biological systems still does not meet the needs, therefore, there has been increasing interest in modifying HPLC, for example, by using smaller column internal diameters, smaller particles or shorter columns. Therefore, ultra-performance liquid chromatography (UPLC), which is considered the most recent advancement in analytical instrumentation, has been dramatically and extensively employed for the analysis of herbal medicines. Based on the narrower columns used in UPLC, a remarkable increase in resolution, sensitivity, and speed of analysis has been achieved. Huang et al. have adopted this separation technique to characterize the absorptive properties of HBA in the intestines [54]. Our laboratory also employed a UPLC system with a 1.8 µm particle diameter column for rapid and sensitive analysis of parishin and its metabolites in rat plasma [60]. Additionally, due to the fact that ppm, even ppb levels of analytes can exerts effects in the extremely high level biological matrix, a good separation of the analyte from the biological matrix is deemed necessary, but the detection system is also fairly important for trace analysis of compounds in a complex biological matrix.

### 3.2. Ultraviolet (UV) Spectroscopy

Numerous types of detection systems are available for LC. The most common one is the UV detector, which is widely used in pharmaceutical analysis due to its economical, practical and availability in most laboratories. Therefore, the UV detector is the preferred choice when possible. The detection wavelength of GAS and HBA is about 220 nm, and the reported lowest limit of quantitation (LOQ) of GAS and HBA is between 10 ng/mL to 20 µg/mL [47,53], so in some cases there might not be enough to determine the analytes in the biological system due to their trace concentration and a more sensitive detector may be needed in analyzing the constituents of *G. elata* in biological samples.

### 3.3. Mass Spectrometry (MS)

With the rapid development of atmospheric pressure ionization MS, including electrospray (ESI) and atmospheric pressure chemical ionization (APCI) in selected ion monitoring (SIM) or multiple reaction monitoring (MRM) mode, MS is considered the most versatile, sensitive and selective technique for trace analysis of compounds in biological samples. Multiple publications have reported trace level analysis of *G. elata* or its constituents using a MS detector. Zhang et al. reported a reproducible and rapid LC-MS method for pharmacokinetic studies of GAS and/or HBA in rat plasma after oral administration of GAS [38], Tianma extract [32] and TianShu capsule [61], which showed a wide linear region with 2.00–200.00 µg/mL for GAS and 0.832–104.00 µg/mL for HBA, and relatively high sensitivity with the LOQ of 25 ng/mL and 50 ng/mL for GAS and HBA.

### 3.4. MS/MS

In recent years, there has been a rapid increase in the number of applications of MS/MS for detection of *G. elata* and its metabolites in biological samples because it allows for collection of both qualitative and quantitative information and provides high sensitivity. With the development of various data acquisition methods, quadrupole-time of flight mass spectrometry (Q-TOF MS) has exhibited excellent performance for metabolite detection because of its high accuracy and high sensitivity. In our previous study, Q-TOF MS was employed to determine the metabolic profile of parishin, NHBA and the ethyl acetate fraction of *G. elata* in vitro and in vivo [49,62,63]. Ni et al. also used Q-TOF MS technology to identify the metabolites of GAS in rat blood after intragastric administration of the extract of Da Chuan Xiong Fang [64]. In addition to the identification of metabolites, the quantitative analysis of the prototype and metabolites or pharmacokinetics of *G. elata* in biological samples, including GAS, HBA, parishin, NHBA, etc., in rat or healthy humans, was widely carried out by the MS/MS technique [65,66,67,68]. The reported limit of detection/quantitation (LOD/LOQ) using the MS/MS technique could reach 0.01 ng/mL for GAS, 2 ng/mL for HBA, 0.083 ng/mL for parishin, and 0.1 ng/mL for NHBA, which represents a quite high sensitivity, satisfying the needs of pharmacokinetic studies. However, the mass spectrometers are quite expensive and not available in many laboratories and in clinic applications, so our laboratory has developed a simple, rapid and sensitive fluorescence detection method to study *G. elata* and its constituents in biological systems.

### 3.5. Fluorescence Detection (FLD)

According to the structures of GAS, HBA, parishin, parishin B and parishin C, the main constituents in *G. elata*, our laboratory reasoned that these constituents may have natural fluorescence properties. Therefore, we verified their natural fluorescence by fluorescence spectroscopy analysis. As we all know, not all compounds display fluorescence properties, and only a fraction of compounds with a specific structure possess such properties. Therefore, FLD provides high sensitivity and selectivity with less matrix interference in biological sample analyses. Consequently, we developed a rapid and sensitive method for the simultaneous determination of parishin and its four metabolites by combined use of UPLC and FLD. The results showed that the LOD of analytes could reach as low as 1 ng/mL in a total run time of 10 min and high selectively with a matrix effect between 93.16% and 104.97%. This method was proved quite robust and suitable for use in the pharmacokinetic study of parishin, parishin B, parishin C, GAS and HBA [60,69,70].

The information of the abovementioned methods for the determination of the constituents of *G. elata*, including LOD/LOQ, detection wavelength (λ)/ion source, run time and type of biological sample, is listed in Table 1.

## 4. Pharmacokinetic Studies

Even though the technical issues in qualitative and quantitative analysis have been overcome to some extent, some common issues in ADME research, such as the design of sampling time points and the number of the test animals, is still challenging because of the complex and unknown natural products in herbal medicines that exhibit various ADME profiles. The sampling time points should be statistically appropriate and adequate enough to ensure the simultaneously ADME profile of multi-compounds in a single in vivo ADME study of an herbal medicine. According to the reported experiments, the sampling time points of *G. elata* or its constituents in animal or human experiments was always in the range of 5 min–24 h. After sampling, the ADME profile of *G. elata* or its constituents was conducted.

### 4.1. Absorption

#### 4.1.1. GAS

The majority of publications showed that the plasma concentration–time curves of GAS after intragastric administration produced one peak, and no enterohepatic circulation was observed [44]. However, Zhang et al. reported that GAS increased rapidly after administration, then decreased and further increased again, giving a second peak in plasma, which suggested that GAS underwent hepatobiliary excretion [32,53]. In addition, according to our statistics, 12 publications showed that the plasma concentration–time curves of GAS was fitted with a two-compartment open model, nine and two publications, respectively, employed a non-compartment open model and one-compartment open model to analyze the pharmacokinetic parameters of GAS [35,40,43]. In addition, Liu et al. reported one-compartment open model for intragastric administration and two-compartment open model for intravenous administration of GAS [71]. GAS was absorbed into the blood stream and reached a peak concentration (C_max_) in plasma at the range of 2–108.5 min after administration of GAS, GAS capsule or *G. elata* extract to SD, Wistar or Kunming rats. Among them, most were lower than 30 min suggesting the rapidly absorption of GAS. Li et al. reported that GAS reached a peak concentration at 90–109.8 min after oral administration to dogs [43]. For healthy administered male humans, the peak time was about 48–49 min [30,42], which was between the data of rats and dogs, suggesting that the absorbed rate of GAS was: rats > human > dog. The absolute bioavailability of GAS was quite high, reaching about 86.1% [31].

#### 4.1.2. *p*-Hydroxybenzyl Alcohol

HBA is the aglycone of GAS according to its structure, so HBA is always detected in biological samples based on the administered GAS or *G. elata* extract. Noteworthily, HBA was detectable only during a time ranging from 30 min to 4 h after administration of GAS at low concentrations in dog plasma samples [66]. In addition, in rat plasma HBA only could be detected at 45 min, 70 min, 100 min and 120 min after oral administration of Tianma extract [32], which might be due to the very low content of HBA present. In the bile and brain, HBA was found at the first sampling point (10 min), declined rapidly after intravenous administration of GAS, and reached a peak concentration in both the brain and bile at 15 min [65]. Interestingly, Cai et al. reported that the concentration-time profile of HBA in brain tissue showed double peaks (t_max1_ = 15 min, t_max2_ = 90 min) [45]. Additionally, Huang et al. used in vitro everted gutsac model and in situ rat single-pass intestinal perfusion model to evaluate the absorption characteristics of HBA in different intestinal segments. The results showed that HBA can be well absorbed via passive diffusion in the intestine, and the absorption rates in the different intestinal segments show no regioselectivity [54].

#### 4.1.3. Parishin

Only one publication systematically reported the bioavailability of parishin, and the authors pointed out that different administered drug styles gave different absorptive properties. When *G. elata* extract was used as the administered formulation, parishin reached a peak time (T_max_) at 38.33 min, 56.67 min and 113.33 min at low, middle and high doses, respectively, whereas, when *G. elata* powder was orally administered, the parishin reached the T_max_ at 26.67 min, 75.00 min and 83.33 min, respectively. The values of T_max_ obtained in middle and high doses after oral administrations of *G. elata* extract were shorter than that obtained from *G. elata* powder, indicating that parishin was absorbed faster when *G. elata* extract was used. The relative bioavailability of parishin from *G. elata* extract to that from *G. elata* powder at low, medium and high dosages were 76.06, 144.08 and 127.75, respectively [34]. In addition, compared with GAS, the peak concentration (C_max_) of parishin was much lower than that of GAS at all three doses, indicating that the absolute bioavailability of parishin was lower than that of GAS. The above research mainly studied the absorption of parishin after administration of crude drugs, whereas our laboratory mainly investigated the pharmacokinetics of monomer parishin and its metabolites by preparation of g-levels of parishin, parishin B, parishin C, parishin E etc., using HPLC. Our results showed that parishin was rapidly metabolized to GAS, HBA, parishin B and parishin C, which all were detectable 5 min after the administration, but we did not detect the protype in the plasma samples. GAS reached a peak concentration of 6.3 µg/mL at 45 min. HBA, parishin B and parishin C reached the peak concentrations of 28 ng/mL, 955 ng/mL and 592 ng/mL at 60 min, 60 min and 30 min, respectively [60].

#### 4.1.4. *N*^6^-(4-Hydroxybenzyl) Adenine Riboside

Our previous studies reported that NHBA could also be rapidly absorbed and reach the peak concentration at 69 min, but the C_max_ was only 107.53 ng/mL at the administered dose of 200 mg/kg body weight, indicating low absolute bioavailability of NHBA [63]. In addition, a NHBA analogue, YZG-331 was also studied. A comparison between the area under the curve (AUC) of YZG-331 obtained after oral and intravenous treatment revealed good absorption of YZG-331 with the bioavailability of 56.8% [68].

### 4.2. Distribution

#### 4.2.1. GAS

In earlier studies, some reported that GAS could not go through the blood brain barrier (BBB), which might be due to the low concentration of GAS in brain tissue and the poor detection limit of the technology employed. With the development of more advanced instrumentation, researchers have reported that GAS could also go across BBB [32], the brain-to-blood distribution ratio of GAS in the brain at doses of 100 mg/kg and 300 mg/kg body weight were 0.007 ± 0.002 and 0.01 ± 0.002 with no significant difference, suggesting a poor BBB penetration of GAS [53], which might be due to the hydrophilic characteristics of GAS. In addition, studies on the distribution of GAS in brain regions showed that the entry of GAS into the brain was rapid, and the levels rapidly declined after drug administration. However, the ratios of AUC_brain_/AUC_plasma_ were not high. The individual ratios of the AUC in the cerebrospinal fluid, frontal cortex, hippocampus, thalamus and cerebellum to the AUC in the plasma were 4.8 ± 2.4%, 3.3 ± 1.2%, 3.0 ± 0.7%, 3.3 ± 1.3% and 6.1 ± 1.9%, respectively. The AUC in the cerebellum was significantly higher than that in other brain regions (*p* < 0.05) [47], which suggested that GAS might have a more potent effect on the cerebellum. The relevance between distribution and the pharmacological effect of the drug in the brain needs further studying.

In addition, Zheng studied the tissue distribution of GAS and parishin after oral administration of extract and power of *G. elata* in rat. The distribution of GAS and parishin in heart, liver, spleen, lung and kidney after oral administration of 4 g/kg body weight powder and 600 mg/kg body weight extract (equal to 4 g/kg body weight of *G. elata*), was studied and the concentration in lung and kidney was found to be higher. Parishin, but not GAS, could be detected in the brain. In the high dose group, GAS and parishin were both detected in the brain, and the cumulative effect was found in kidney and lung. Additionally, the tissue distribution of GAS and parishin after oral administration of extract and power of *G. elata* were similar [72].

#### 4.2.2. *p*-Hydroxybenzyl Alcohol

After administration of GAS, HBA formed immediately, and then passed through the blood–brain barrier and produced a pharmacological effect. However, the concentration was low and declined very quickly in the cerebrospinal fluid and plasma [47].

### 4.3. Metabolism and Biotransformation

Ideally, drug metabolism should be studied before the registration of a bioactive drug. Hepatic cytochrome enzyme act as a key role in phase I and phase II xenobiotic metabolism. Drugs with enzyme action can result in drug metabolism of active or toxic substances, exerting subtherapeutic drug concentrations or showing drug accumulation and drug toxicity. Therefore, drug metabolism is an indispensable process in the development of drugs.

#### 4.3.1. Phenols

Lu et al. were the first to report that GAS could be metabolized to HBA in vivo [45,73,74]. After that, they further studied the hydrolyzation of GAS in kidney, liver, brain and plasma. The intensity of hydrolysis in tissues was as follows: kidney > liver, brain. In addition, two other metabolites of GAS, *p*-hydroxybenzoic acid and *p*-hydroxybenzopyran glucuronic acid, were first detected in urine samples [75]. With the improvement of analytical technology, more metabolites of GAS were detected recently. Two phase I metabolites of GAS, including dehydrocarbyl and demethoxyl products, and two phase II metabolites, including acetylated and combined with amino-acid products, were identified in rabbit plasma after oral administration of Dachuanxiong Decoction extraction [64].

**Scheme 1:**
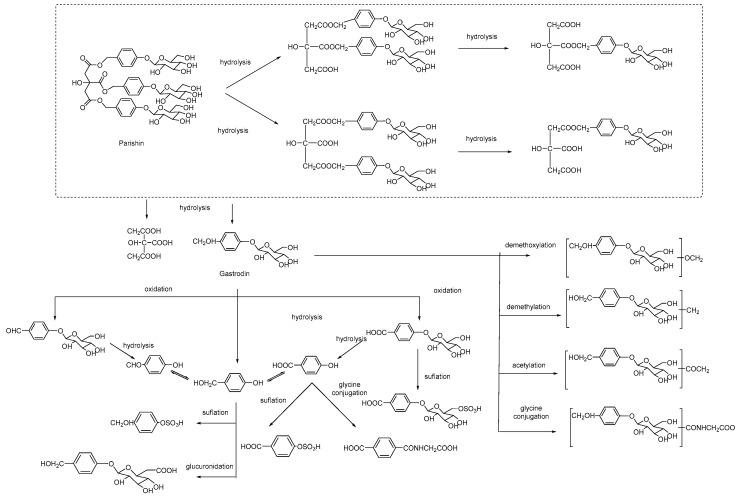
The metabolic pathways of phenolics from *G. elata*.

Additionally, Jia et al. identified five metabolites in rat plasma, including *p*-formylphenyl-β-d-glucopyranoside, *p*-hydroxybenzoic acid, HBA, *p*-formaldehydephenyl-β-d-glucopyranoside, *p*-hydroxybenzaldehyde. Other than HBA, all others were all first detected in rat plasma samples [36]. Furthermore, we reported the metabolic process of GAS and parishin and showed that seven metabolites of GAS, 14 metabolites of parishin (seven hydrolyzates and seven derivatives of GAS) were detected and identified in rat plasma and urine after intragastric administration [49]. We further studied the metabolic process of phenolics in *G. elata*, and detected 24 metabolites in rat plasma samples after intragastric administration of the ethyl acetate fraction of *G. elata* (2.5 g/kg body weight) [62]. According to the structure of metabolites from *G. elata* or its main constituents, we summarized that the main metabolic process of phenolics of *G. elata* are glucuronidation, glucosylation, sulfation, methylation, hydroxylation, dehydrogenation or mixed modes, and the main metabolic pathways is shown in Scheme 1.

#### 4.3.2. *N*^6^-(4-Hydroxybenzyl) Adenine Riboside

**Scheme 2:**
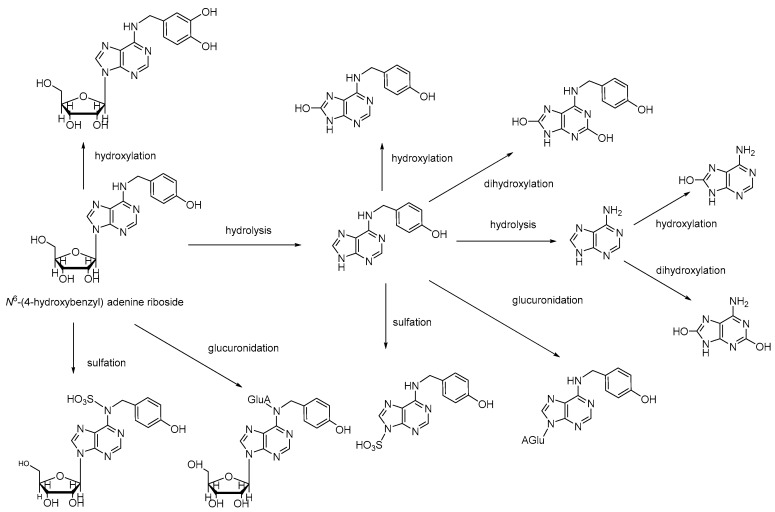
The metabolic pathways of *N*^6^-(4-hydroxybenzyl) adenine riboside from *G. elata*.

The metabolites in rat urine and plasma of NHBA were analyzed by HPLC-ESI-MS/MS after oral administration (100 mg/kg body weight). Six phase I metabolites and four phase II metabolites were identified in urine samples and proved to be mainly formed via hydrolysis or hydroxylation in phase I reaction, *N*-sulfation or *N*-glucuronidation in phase II reaction or their combinations. Among them, one phase I metabolite and two phase II metabolites were also identified in rat plasma [67]. Given that the amount of metabolites in plasma was near the detection limit, some metabolites might not be detected, the metabolic behavior in vitro of NHBA was studied by our laboratory. Two metabolites were detected and identified as *N*^6^-(4-hydroxylbenzyl) purine and *N*^6^-(3,4-dihydroxylbenzyl) adenine riboside by UPLC-QTOF/MS and NMR technology, and these two metabolites showed high neuroprotective action [76], suggesting that the metabolites might be the real pharmacological active substances in vivo. Therefore, except for the abovementioned two metabolites, the others could be further explored for their phamacological efficacy to discovery new therapeutical drugs. We summarize the main metabolic pathways of NHBA according to our studies, in Scheme 2.

### 4.4. Excretion

Lu et al. reported that a very high proportion of unchanged GAS is excreted in the urine, and a low proportion of GAS undergoes biliary excretion. The bile-to-blood coefficients of unbound GAS following 100 mg/kg and 300 mg/kg body weight of GAS administration were 0.14 ± 0.02 and 0.18 ± 0.02, which might be due to its low molecular weight which is less than 300 [45,53]. The elimination half life (t_1/2_) of GAS was at the range of 8.41–181.16 min after administration in rat, whereas for *G. elata* extract administered to rat, the average t_1/2_ was higher, and could even reach 345 min, indicating that other constituents in *G. elata* extract have an effect on the excretion of GAS, delaying the excretion. In rabbit, the t_1/2_ of GAS was about 38–47 min, which was similar to that in rat. Additionally, the t_1/2_ in healthy male humans was quite high, which could reach 226.8–330 min, suggesting the elimination rate of GAS in humans was relative slower than in rats and rabbits. Based on the above discussions and guidance from related publications, we can conclude that the pharmacokinetic studies of *G. elata* was always concentrated on the single active compounds in *G. elata*. It is well known that herbal medicines are a quite complex system with many active constituents. Many of them might be absorbed into the blood after dosing, exerting favorable pharmacokinetic properties and certain efficacy. Therefore, the pharmacokinetic properties of one active compound alone cannot represent the whole pharmacokinetic behavior and consequently, a strategy for an integrated pharmacokinetic study of *G. elata* is proposed in our laboratory. The major objectives of this strategy were to comprehensively characterize the holistic pharmacokinetic behavior of *G. elata*, which could provide guidance for dosage design and clinical medication to some extent. Considering the structural similarity of many constituents, the integrated pharmacokinetics mainly based on an AUC-weighing approach [77,78], which was proposed to describe the holistic pharmacokinetic profile of parishin, *G. elata* extract and Rhizoma Gastrodiae capsule [69]. The integrated bioavailability of parishin was calculated as 13.84%. In addition, for the administered *G. elata* extract, the exposure level in vivo of HBA, parishin B and parishin C was quite lower than that of GAS. Therefore, GAS could reveal the overall pharmacokinetic properties of *G. elata* extract, while for administered Rhizoma Gastrodiae, HBA also occupied approximately 30% exposure level in vivo, so integrated data of GAS and HBA might be more suitable to show the holistic properties. The pharmacokinetic information of *G. elata* or its constituents is summarized in Table 2.

### 4.5. Influencing Factors

#### 4.5.1. Routes of Administration and Dosage

Different modes of administration (intravenous injection, intragastric administration and intranasal administration, etc.), dosage of administration and type of drug all have certain impact on the pharmacokinetic properties of *G. elata*. Wang et al. reported that the pharmacokinetic properties of GAS after duodenal administration were similar to those obtained by intravenous administration, and the intranasal administration of GAS provided a comparable AUC in cerebrospinal fluid (CSF) compared with the intravenous administration. However, the GAS level in plasma was very slow via intranasal administration. The ratios of AUC values of intranasal to intravenous administration were 8.8% and 105.5% in plasma and CSF, respectively [57]. Zhao et al. reported that the relative oral bioavailability of GAS and parishin were higher when administrated with *G. elata* powder at low dosage. In contrast, the relative oral bioavailability of GAS and parishin was higher when administrated with *G. elata* extracts at medium and high dosages [34].

#### 4.5.2. Compatibility

There have been five reports about the influence of compatibility on the pharmacokinetics of GAS in different formulas. One study examined the influence of the compatibility of ophiopogonis tuber and Chinese magnoliavine fruit with *G. elata* on the pharmacokinetics of GAS in rat, three dosages of Tianma granule extract (equivalent to GAS 50, 100, 200 mg/kg body weight) and one dosage of Tianma extract (equivalent to GAS 100 mg/kg body weight) were separately administered to rats by intragastric administration. The results showed that the compatibility of ophiopogonis tuber and Chinese magnoliavine fruit with *G. elata* can delay the absorption, reduce the elimination rate and prolong the action time of GAS in vivo [40].

Zheng et al. focused on determining the influence of the compatibility on pharmacokinetics of GAS in different prescription proportions of *G. elata* and Chuanxiong (1:0; 1:0.25; 1:2.1; 1:4.2). The results showed that the pharmacokinetic parameters, AUC and C_max_ of GAS were dramatically different after oral administration of *G. elata* and the different combinations of its constituent herbs. Chuanxiong had some retarding influence on the absorption, distribution and excretion of GAS in vivo [35]. Hu et al. also studied the possible pharmacokinetic behavior differences of GAS after individually oral administration of Tianma extract and Tianma extract mixed with different active ingredients of Chuanxiong to rats, as well as explored whether there existed some herb-herb interactions. The results showed that tetramethylpyrazine had no significant effect on the pharmacokinetic parameters of GAS (*p* > 0.05), whereas ferulic acid, total phenolic acids and total alkaloids significantly increased AUC_0–∞_ (*p* < 0.05) [37]. Interestingly, Gouteng was also reported as having similar influence on GAS in vivo as Chuanxiong. The rationale of the compatibility of Gouteng and *G. elata* as a classic “herb pair” was verified from the point of pharmacokinetics [41]. In addition, Jiang et al. reported that GAS has a higher bioavailability and lower clearance rate, as well as longer mean residence time both through intragastric and intravenous routes, particularly notable via intragastric administration than those given alone. The relative oral bioavailability of GAS in combination administration is 1.5-times greater than that of a single administration [52]. *G. elata* is a common used medicine in Chinese medicine formulae, many formulaes contain *G. elata*, such as Angong Jiangya Wan, Tiandan Tongluo Pian, and so on. Formulae are the main clinical patterns of *G. elata* and the core of formulae is prescription compatibility. Thus prescription compatibility becomes a critical issue in *G. elata* study. In addition, the differences in pharmacokinetic properties of *G. elata* also note the importance of investigating the pharmacological characteristics of *G. elata* prescriptions used in clinical applications. Considering the fact that composition of relevant *G. elata* prescription is complex, most of scholars have only studied the interactions of effective monomer component. With the appearance of integrated pharmacokinetics, multi-components interactions might receive extensive attention.

#### 4.5.3. Food

Many natural products exhibit a background baseline in biological fluids due to their occurrence in the diet. Jia et al. reported the influence of multiple-dosing and food on the pharmacokinetic behaviors of GAS and its metabolites. Food in single dose groups had a great impact on pharmacokinetic parameters of GAS and all of the metabolites. All compounds can be found about half decrements of C_max_, AUC and t_1/2_ in the fed group than the fasted group; while no significant difference of t_max_ was found. In multiple-dose groups, co-administation with food did not have a great influence on the pharmacokinetic behavior as it did in single-dose conditions, which was similar to that in the single-dose fed group. Altogether, the results suggest administration of GAS in the fasting state is optimal for much better absorption, regardless of single-dose groups or multiple-dose groups [36]. This suggests that taking GAS by way of the gastrointestinal tract will result in a quicker plateau concentration if taken on an empty stomach.

## 5. Summary and Perspectives

Although *G. elata* has been used for thousands of years in China, the lack of standardization and safety and efficacy studies vastly restricts its utilization worldwide. Our intention is to encourage more research from the pharmacokinetic viewpoint in order to evaluate the potential of *G. elata*, or development of its specific components as a novel therapeutical drug. Since ancient times, GAS and HBA have been widely considered to be the major bioactive constituents or metabolites of *G. elata* in vivo. With phytochemical studies, more constituents, such as parishins and hydroxybenzyl nucleosides, have been reported to exert a greater potential effect. One challenge is the poor lipid-solubility and low bioavailability of the parishins, which might due to the multiple sugar moieties in the structure. Chen et al. reported that the influence of sugar moieties on the absorption and metabolism of *Epimedium* flavonoids in vitro, the result showed that icariin (diglycosides) and epimedins A–C (triglycosides) could not be directly absorbed. They firstly need to hydrolyze to a monoglycoside, and the hydrolyzed metabolite could be subsequently absorbed [79], which means that multiple sugar moieties obstruct the absorption of compounds. Therefore, the chemical modification approaches or pharmaceutical approaches through nanoparticle- or lipid-based delivery formulation may help to improve the delivery and bioavailability of the parishins. In addition, the future will see the increasing application of multi-component pharmacokinetic therapy in herbal medicine. The recent integrated pharmacokinetic approach offers an alternative to address the challenges of the determination of PK of multi-component herbal medicines. Nowadays, the integrated mode is mainly based on the structure analogues in vivo, for compounds with vastly different structure, and there are no good solutions. Therefore, putting forward much more reasonably integrated modes is essential. Additionally, the integrated pharmacokinetic parameters of multi-component herbal medicine is essential to reduce overdosing and drug complications, keeping healthcare costs at a minimum, increasing patient compliance and quality of life.

Additionally, the present research on pharmacokinetics is only limited to the influence on the in vivo dynamic processes of certain ingredients; that is, there is a lack of studies on in vivo drug interaction of main efficacious components in the course of ADME, which is helpful to illustrate the principle of pharmacokinetic compatibility from the essence. Considering the fact that the composition of TCMs is complex, the quality is hard to control, and the therapeutic mechanisms are difficult to explore, some scholars have only studied the interactions between effective monomer components. Moreover, most publications focused on the normal subjects, including rat, dog, human etc., and further studies are necessary to elucidate the pharmacological and pharmacokinetic properties of *G. elata* and its constituents under pathological conditions, such as brain injury, which might be quite different from a normal state.

## Figures and Tables

**Table 1:** The main method and relative parameters for the determination of constituents of *G. elata*.

Number	Method	Analytes	Type of Biological Sample	Time (min)	LOD/LOQ (ng/mL)	λ (nm/ESI)	Ref.
1	HPLC-UV	GAS and HBA	Plasma, CSF and Brain microdialysate	<30	280, 160 and 70/150, 70 and 40	221	[47]
2	UPLC-UV	HBA	Duodenum, jejunum, ileum and colon	<2	-	220	[47]
3	HPLC-ESI/APCI-MS	GAS and HBA	Plasma	<10	2000 and 830	(−)	[32]
4	HPLC-ESI-MS	GAS and HBA	Plasma	<30	500	(−)	[41]
5	HPLC-ESI-MS/MS	GAS	Plasma and dialysates	<15	0.01	(+)	[48]
6	HPLC-ESI-MS/MS	GAS	blood, brain and bile	<10	0.5	(−)	[65]
7	HPLC-ESI-MS/MS	HBA	blood, brain and bile	<10	0.2	(−)	[65]
8	UFLC-ESI–MS/MS	GAS and its five metabolites	Plasma	<11	1	(−)	[36,66]
9	HPLC-ESI-TSQ/MS	YZG-331	Plasma	<7	25	(+)	[68]
10	UHPLC-ESI-TSQ/MS	Parishin	Plasma	<10	0.083	(−)	[34]
11	UPLC-QTOF/MS	NHBA	Plasma	<5	0.1	(+)	[63]
12	UPLC-FLD	GAS, HBA, PB, PC, PA	Plasma	<10	0.6, 0.8, 1, 1, 1	λ_ex_: 225 λ_em_:295	[60]

**Table 2:** Pharmacokinetic information of *G. elata* or its constituents.

Target	Subject	Administered Route/Dosage (mg/kg)/Tissue	T_max_ (min)	C_max_ (μg/mL)	t_1/2_ (min)	AUC (μg·min/mL)	CL, CL/F (mL/min/kg)	V, V/F (mL/kg)	MRT (min)	Ref.
GAS	Rabbit	iv/100, iv/200	-	-	t_1/2α_: 5.2, t_1/2β_: 38.1	14,356	6.98	387	-	[73]
	Rat	iv/100	-	-	t_1/2α_: 0.49, t_1/2β_: 8.41	3200	38	450	-	[74]
		iv/200	-	-	t_1/2α_: 1.9, t_1/2β_: 12.2	7900	25.3	442	-	[74]
		iv/200	-	-	t_1/2α_: 4.6, t_1/2β_: 35	21,000	-	-	-	[31]
		ig/200	48	140	t_1/2α_: 22, t_1/2β_: 52	18,000	-	-	-	[31]
		ig/200 bile duct ligation	64	110	t_1/2α_: 27, t_1/2β_: 60	15,000	-	-	-	[31]
		iv/100	-	533	38.8	8601	13.3	215	17.4	[53]
		iv/300	-	1033	38.2	29,882	102	237	23.2	[53]
		in/50	-	-	-	222	-	-	-	[46]
		iv/50	-	-	-	2597	-	-	-	[46]
		iv/200	-	350.9	41.2	19,019	-	-	57.5	[47]
		ig/20,000	70	13.48	-	1870	4.28 mL/min	0.27 L	-	[32]
		ig/200	5–30	128.4–209.8	-	16,297–20,174	-	-	104.8–115.2	[33]
		ig/50	21.00	29.75	172.64	3245	15.68	3710.74	-	[39]
		ig/100	34.50	42.80	181.16	5139	19.22	4872.31	-	[39]
		ig/200	108.50	30.85	109.67	8593	23.59	3776.81	-	[39]
		ig/50 granule	26.88	18.56	239.34	3454	14.93	4974.58	278.78	[40]
		ig/100 granule	42.13	28.40	225.60	5901	18.07	5808.72	337.13	[40]
		ig/200 granule	36.00	25.45	205.71	6698	31.22	9235.03	377.37	[40]
		ig/100	34.50	42.80	181.16	5441	19.22	4872.31	171.85	[40]
		ig/20	13.4–18.5	-	t_1/2α_: 2.1–9.1, t_1/2β_: 24.8–167.9	228–280	-	-	-	[35]
		ig/9 g/kg	45	140.72	-	13,816	3.50	-	161.69	[41]
		ig/5.4 g/kg	21.67	188.45	-	21,550	2.32	-	121.74	[41]
		ig/315 extract	18.33	1.05	326.35	96	2570	1,196,580	-	[34]
		ig/630 extract	12.50	2.97	176.51	216	2720	701,130	-	[34]
		ig/1260 extract	14.17	4.66	158.82	293	4030	920,850	-	[34]
		ig/315 powder	18.33	1.25	177.70	131	2180	574,840	-	[34]
		ig/630 powder	25.00	1.25	345.07	113	4380	2,152,710	-	[34]
		ig/1260 powder	40.83	2.29	260.50	229	5280	2,164,030	-	[34]
		ig/100	25.2	44.84	67.8	3475	29.67	2920	60.18	[70]
		ig/116	49.8	3.47	185.4	298	393.33	105,020	88.2	[70]
		ig/2300	60	14.18	451.2	2183	2147.67	1,415,580	128.4	[70]
		iv/72.5	-	-	67.2	1560	48.33	3720	-	[69]
		iv/116	-	-	70.2	1850	64	6450	-	[69]
		iv/220	-	-	73.2	5108	43	4450	-	[69]
		iv/64.5	-	-	78.6	3181	20.33	2370	-	[69]
		ig/1 g/kg	40.2	0.26	60.6	18.6	56,293.17	4,139,720	90	[69]
		ig/4 g/kg	42	0.21	69.6	69	58,714.17	7,270,960	109.8	[69]
		ig/40	15–33	18.43–21.74	58.2–168.6	1039–1630	24.54–40.43	-	49.8–82.2	[52]
		iv/20	-	69.97–75.65	68.4–102	1324–1634	15.13–12.20	-	21.6–27.6	[52]
		ig/21	22.8	10.78	-	557	-	-	71.4	[71]
		iv/21	78	-	-	1651	-	-	64.8	[71]
		ig/0.322 g	12–15	1.67–4.34	t_1/2α_: 9.3–29.4, t_1/2β_: 51.3–235.9	233–372	0.01–0.02 L/min	0.348–1.424 L	122.3–290.9	[37]
		iv/100/Brain	-	1.4	14.6	42	-	-	24.7	[53]
		iv/300/Brain	-	5.2	58.4	353	-	-	82.5	[53]
		ig/200/Brain	5–15	0.45–0.79	-	50–105	-	-	77.56–139.68	[33]
		iv/200/CSF	15.0	16.1	64.7	848	-	-	66.4	[47]
		iv/200/Frontal cortex	-	21.6	29.6	588	-	-	26.0	[47]
		iv/200/Hippocampus	-	24.3	25.6	580	-	-	22.7	[47]
		iv/200/Thalamus	-	22.0	27.8	570	-	-	27.9	[47]
		iv/200/Cerebellum	-	35.8	25.2	1042	-	-	26.8	[47]
		iv/100/Bile	-	37.1	27.6	1048	-	-	38.1	[53]
		iv/300/Bile	-	214	34.6	5352	-	-	30.8	[53]
	Dog	ig	90	51.0	t_1/2α_: 2.49, t_1/2β_: 6.43	40,494	-	-	-	[43]
		ig/40	109.8	23.00	t_1/2α_: 111.6, t_1/2β_: 288	7230	7.16	0.0016	-	[66]
		iv/50	-	-	t_1/2α_: 4.64, t_1/2β_: 105	3800	1.4	210	-	[74]
	Human	ig/200	48.6	1.48	t_1/2α_: 226.8, t_1/2β_: 363.6	337	-	-	-	[30]
		ig/20/experiment	48.6	1.48	330	344	-	-	-	[42]
HBA	Rat	ig/9 g/kg	55	10.45	-	2267	-	-	266.67	[41]
		ig/5.4 g/kg	60	9.12	-	1656	-	-	154.12	[41]
		ig/200/Brain	T_max1_:15 T_max2_: 90	C_max1_: 0.55 C_max2_: 0.71	54.8	52,823 ng min/g	-	-	-	[45]
		iv/50	15	0.11	-	-	-	-	-	[65]
Parishin	Rat	ig/315 extract	38.33	0.02	53.24	1.84	0.18	13.44	-	[34]
		ig/630 extract	56.67	0.030	62.42	4.64	0.14	12.32	-	[34]
		ig/1260 extract	113.33	0.031	38.49	4.59	0.27	15.29	-	[34]
		ig/315 powder	26.67	0.017	62.48	2.42	0.16	16.35	-	[34]
		ig/630 powder	75.00	0.021	54.25	3.22	0.24	21.28	-	[34]
		ig/1260 powder	83.33	0.021	54.28	3.60	0.15	31.2	-	[34]
		iv/72.5/	-	-	21.6	268	278.83	8680	-	[69]
		iv/116/	-	-	17.4	358	337.5	8840	-	[69]
		iv/220/	-	-	24.6	1057	295	12,630	-	[69]
Integrated parishin	Rat	iv/72.5	-	-	72.6	1118	64.83	6930	-	[69]
		iv/116	-	-	76.8	1387	83.5	9290	-	[69]
		iv/220	-	-	66.6	3629	60.5	5830	-	[69]
		ig/116	-	-	70.2	192	599.17	60,990	89.4	[69]
Integrated Gastrodiae capsule	Rat	ig/1 g/kg	-	-	80.4	15.6	63,881.17	7,411,560	91.2	[69]
		ig/4 g/kg	-	-	91.8	54.6	70,464.67	93,335,990	160.2	[69]
NHBA	Rat	ig/200	69	0.11	465	26	6619.33	5,340,000	486	[63]

T_max_: time reached the maximum concentration; C_max_: maximum concentration; t_1/2_: elimination half-life; AUC: area under the curve; CL: clearance; V: apparent volume of distribution; MRT: mean residence time; F: bioavailability; ig: intragastrical administration; iv: intravenous administration; in: intranasal administration; CSF: cerebrospinal fluid.

## Supplementary Materials

Supplementary materials are available online. Figure S1: the total ion chromatograms of gastrodin and parishin in 10% methanol-water (A), gastrodin and parishin in rat plasma extracted with methanol (B), gastrodin and parishin in rat plasma extracted with C18-SPE (C), gastrodin and parishin in rat plasma extracted with MCI-SPE (D) by UPLC-QTOF MS.
