# Supplementary material for: Analytical Techniques and Pharmacokinetics of Gastrodia elata Blume and Its Constituents
Source: Molecules. 2017 Jul 8;22(7):1137. doi: 10.3390/molecules22071137 (PMC6152015; doi:10.3390/molecules22071137)
Supplement: Supplementary file 1 [file molecules-22-01137-s001.pdf]

Analytical Techniques and Pharmacokinetics of *Gastrodia elata* Blume and Its Constituents

Jinyi Wu, Bingchu Wu, Chunlan Tang* and Jinshun Zhao*

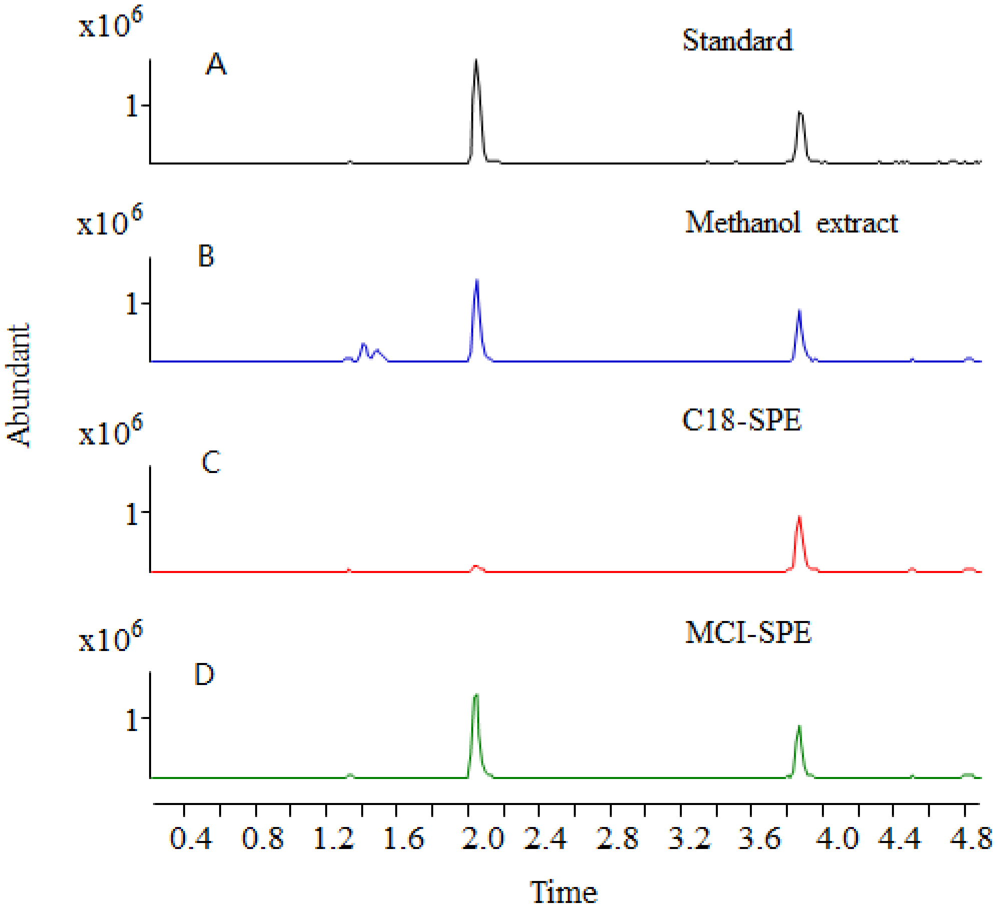

Fig.S1: The total ion chromatograms of gastrodin and parishin in 10% methanol-water (A), gastrodin and parishin in rat plasma extracted with methanol (B), gastrodin and parishin in rat plasma extracted with C18-SPE (C), gastrodin and parishin in rat plasma extracted with MCI-SPE (D) by UPLC-QTOF MS.
